# Does timing of systemic antibiotics influence periodontal treatment outcomes? A randomized clinical trial

**DOI:** 10.1002/jper.70057

**Published:** 2026-02-07

**Authors:** Daiane Fermiano, Eduardo de Souza Oliveira, Maria Josefa Mestnik, Luciene C. Figueiredo, Takahiko Shiba, Shunsuke Matsumura, Edson de Sousa, Helio Doyle, Rafael Oliveira Dias, Marcelo de Faveri, Magda Feres

**Affiliations:** ^1^ Dental Research Division Guarulhos University Guarulhos Brazil; ^2^ Department of Oral Medicine, Infection, and Immunity Harvard School of Dental Medicine Boston Massachusetts USA; ^3^ Department of Periodontology, Graduate School of Medical and Dental Sciences Institute of Science Tokyo Tokyo Japan

**Keywords:** antibiotics, biofilm, microbial interactions, periodontal debridement, randomized clinical trial, scaling and root planing

## Abstract

**Background:**

The aim of this study is to determine whether the timing of metronidazole (MTZ) and amoxicillin (AMX) administration, relative to scaling and root planing (SRP), influences the clinical and microbiological outcomes of periodontal treatment.

**Methods:**

In this double‐blind, placebo‐controlled, randomized clinical trial, subjects with stage III/IV periodontitis were assigned to receive SRP over 14 days with either (1) MTZ+AMX three times a day (TID) during SRP followed by placebo (*early* antibiotic group) or (2) placebo during SRP followed by MTZ + AMX TID immediately after SRP (*late* antibiotic group). Clinical parameters (probing depth [PD], clinical attachment level [CAL], visible plaque, bleeding on probing, suppuration) were recorded at baseline, 3 months and 1 year. Subgingival biofilm samples from nine sites per subject were individually analyzed using checkerboard DNA–DNA hybridization targeting 40 bacterial species. Bacterial correlation networks were constructed using SparCC and visualized in Cytoscape based on the bacterial abundance.

**Results:**

Sixty‐eight subjects were included in the study, with 34 per group. Both groups achieved comparable clinical improvements. Approximately 60% of participants in each group reached the primary treatment endpoint at 1 year (≤ 4 sites with PD ≥ 5 mm at 1 year; *p* > 0.05), and the reductions in PD and CAL did not differ significantly between groups (*p* > 0.05). Microbiologically, the *early* antibiotic group demonstrated faster and more pronounced ecological reorganization, with reduced centrality of red and orange complex species and earlier integration of health‐associated taxa such as *Actinomyces* spp. In contrast, the *late* group showed slower microbial restructuring, with *Actinomyces* taxa remaining entangled with orange‐complex organisms and persistent dysbiotic clusters dominated by *Fusobacterium* subspecies.

**Conclusions:**

While the timing of antibiotic administration relative to SRP did not influence the overall clinical efficacy of periodontal treatment, initiating antibiotics on the first day of mechanical debridement promoted a more rapid and beneficial restructuring of the subgingival microbial community.

**Plain language summary:**

When treating severe gum disease, dentists often combine deep cleaning with antibiotics to help eliminate harmful bacteria. However, the best time to give these antibiotics—at the start or the end of the cleaning process—has never been tested. This study compared two approaches in 68 patients with advanced gum disease: one group received antibiotics during their cleaning sessions over 2 weeks, while the other received them immediately after cleaning finished. Both groups showed similar improvements in gum health after 1 year, with about over half achieving successful treatment outcomes. However, analyzing the bacterial communities revealed an interesting difference: patients who started antibiotics early experienced faster and more thorough changes in their mouth bacteria. Their harmful bacteria disappeared more quickly, and beneficial bacteria formed a stable, protective community sooner. In contrast, the late‐antibiotic group showed slower bacterial changes, with beneficial bacteria remaining mixed with harmful ones for longer. While both timing strategies work clinically, starting antibiotics at the beginning of treatment may help to establish a healthier, more stable bacterial community in the gums, potentially offering better long‐term protection against disease recurrence.

## 1 INTRODUCTION

Scaling and root planing (SRP) remains the cornerstone of periodontal therapy, aiming to disrupt and remove subgingival biofilm and calculus to re‐establish a biologically compatible environment. However, in patients with generalized and advanced forms of periodontitis, SRP alone often fails to achieve sustained clinical improvements or to promote a stable microbial shift toward a health‐associated profile. This is particularly evident in individuals with high levels of inflammation, deep pockets, or systemic modifiers of disease progression.^1^, ^2^, ^3^

To enhance the clinical and microbiological efficacy of SRP, systemic antibiotics, particularly the combination of metronidazole (MTZ) and amoxicillin (AMX), have been widely investigated as adjuncts.^4^, ^5^, ^6^, ^7^, ^8^ Randomized controlled trials and meta‐analyses consistently demonstrate additional clinical and microbiological benefits from the use of these agents, especially in patients with severe disease.^4^, ^8^, ^9^, ^10^, ^11^, ^12^ Despite this evidence, the protocols for antibiotic use in periodontics remain largely empirical, often borrowed from general medicine rather than grounded in periodontal‐specific pharmacodynamics. As a result, fundamental questions remain regarding the optimal dosage, duration, and timing of administration.

Among these variables, the timing of systemic antibiotic administration in relation to the mechanical disruption of the biofilm may be critical, yet it has been systematically overlooked. To our knowledge, no randomized clinical trial has directly compared different timings of systemic antibiotic initiation relative to SRP. In theory, the two most commonly used strategies—starting antibiotic treatment at the first or last SRP sessions—both have biologically plausible rationales.^9^ Administering antibiotics at the end of the mechanical phase may optimize drug access by first disrupting the biofilm barrier. Conversely, starting the antibiotic at the onset of SRP could enhance systemic and local delivery through increased gingival crevicular fluid and vascular permeability—conditions typically present during the early inflammatory phase. Moreover, the immediate exposure of pathogens to systemic antibiotics may promote a more rapid microbial shift, favoring beneficial recolonization of treated sites.^9^, ^13^, ^14^, ^15^

The present study was designed to test whether timing alone influences the clinical and microbiological outcomes of adjunctive antibiotic therapy in periodontitis. We evaluated the clinical and microbiological outcomes of administering MTZ+AMX either at the beginning (*early* antibiotic group) or at the end (*late* antibiotic group) of the SRP treatment, using a standardized clinical endpoint for disease resolution and microbial shifts. By addressing a clinically relevant yet underexplored aspect of therapy, this study aims to refine the strategic use of systemic antibiotics in periodontal care.

## 2 MATERIALS AND METHODS

### 2.1 Subject population, inclusion, and exclusion criteria

Following approval by the Ethics Committee of Guarulhos University (CAAE: 32465714.4.1001.5506/SISNEP/726), this study was conducted in accordance with the Declaration of Helsinki as revised in 2013. All participants provided written informed consent prior to their inclusion. The trial was registered at ClinicalTrials.gov (ID: NCT06177119).

Inclusion criteria were: diagnosis of stage III or IV, generalized, grade B or C periodontitis,^16^ age 35 years or older; at least 15 teeth (excluding third molars and teeth indicated for extraction); a minimum of six teeth with at least one interproximal site each presenting probing depth (PD) and clinical attachment level (CAL) ≥ 5 mm; and more than 30% of sites with PD and CAL ≥ 4 mm along with bleeding on probing.

Exclusion criteria included: current pregnancy or breastfeeding; current smokers or those who had quit within the past 5 years; systemic conditions known to influence the progression of periodontitis (e.g., diabetes); previous SRP within the last 1 year; antibiotic use within the past 6 months; long‐term use of anti‐inflammatory medication; need for antibiotic prophylaxis before dental procedures; and allergy to MTZ and/or AMX.

### 2.2 Primary and secondary outcome variables

The primary outcome variable was the number and percentage of participants achieving the defined clinical endpoint for periodontal treatment: ≤4 sites with PD ≥5 mm^17^, ^18^ assessed at 1 year post‐therapy.

Secondary outcomes included between‐group differences in changes in CAL, PD, the proportion of sites with visible plaque, gingival bleeding, bleeding on probing (BOP), suppuration (SUP), and the levels, proportions and co‐occurrence network analysis of 40 bacterial species.

### 2.3 Sample size calculation

The ideal sample size to assure adequate power for this study was based on the percentage of volunteers reaching ≤ 4 sites with PD ≥ 5 mm.^18^, ^19^ Considering a difference of 36 percentage points between the treatment groups in the proportion of volunteers achieving this endpoint at 1 year post‐treatment, and a significance level of 5%, it was calculated that a total of 57 individuals (approximately 28 per group) would be necessary to provide a power of 80%. Taking into account a possible attrition rate of 15%, the final sample size was adjusted to 68 participants.

### 2.4 Experimental design, randomization, treatment protocol, and allocation concealment

In this double‐blind, placebo‐controlled, parallel‐arm randomized clinical trial, participants with stage III/IV generalized periodontitis were randomly assigned (using computer‐generated randomization tables, in blocks of four and stratified by therapy) to one of two groups: the *early* antibiotic group or the *late* antibiotic group, as detailed in the antibiotic protocol described below. All participants underwent scaling and root planing (SRP). Following the initial clinical evaluation and biofilm samples collection, participants received supragingival scaling and oral hygiene instructions, followed by 4–6 sessions of SRP utilizing Gracey curettes (#5/6, 7/8, 11/12, and 13/14 from Hu‐Friedy, Chicago, IL, USA), performed under local anesthesia. Each SRP session lasted about 1 h and was conducted over a 14 day period. Periodontal treatment provided during the study was offered at no cost. Additional dental treatment needs identified during the study were referred to the appropriate departments within the dental clinic of Guarulhos University.

Antibiotic protocol: In addition to the SRP treatment, the *early* antibiotic group received MTZ (400 mg) + AMX (500 mg) co‐administered three times a day (TID) during SRP, followed by 14 days of placebo. The *late* antibiotic group received placebos TID during SRP and began the same antibiotic regimen immediately after SRP completion, continuing for 14 days. Patients were seen on day 14 of antibiotic/placebo intake to return the medication bottles and to undergo compliance monitoring.

### 2.5 Monitoring of compliance

A study assistant monitored adherence to the antibiotic/placebo regimen by calling patients three times per week during the medication period. An additional tablet was included in each medication bottle as a compliance strategy to ensure complete ingestion of the prescribed course.

The antibiotics and placebos were prepared (Pharmedica Pharmacy, São Paulo, SP, Brazil) in capsules of identical appearance and packaging. The medications were sent to the study coordinator (M. Fav.), who labeled the bottles according to the treatment allocation list. Allocation concealment was ensured by placing the numbered bottles in sealed, indistinguishable plastic bags bearing only the participant number. All study personnel, including clinicians, examiners, and participants, were blinded to group assignment throughout the trial. Participants received clinical evaluations at baseline, 3 months, and 1 year post‐treatment. In addition, all participants received periodontal maintenance therapy and individualized oral hygiene instructions every 3 months following active therapy.

### 2.6 Clinical monitoring and calibration exercise

Visible plaque, gingival bleeding, BOP, SUP, PD, and CAL were recorded at baseline, and at 3 months and 1 year post‐SRP. Measurements were taken at six sites per tooth (excluding third molars) using a manual periodontal probe (North Carolina—Hu‐Friedy, Chicago, IL, USA). All clinical measurements were performed by a single examiner (DF), who underwent a calibration exercise prior to the study. The examiner achieved a standard error of measurement of 0.21 mm for PD and 0.24 mm for CAL, and demonstrated a 90% agreement rate for categorical variables, as assessed by the weighted Kappa test.

### 2.7 Microbiological monitoring

Nine subgingival sites were selected per participant—three from each of the following categories: shallow (PD ≤ 3 mm), moderate (PD 4–6 mm), and deep (PD ≥ 7 mm). The selected sites were nonadjacent interproximal surfaces distributed across all four quadrants.^20^

After clinical measurements and supragingival plaque removal, subgingival biofilm was collected from each site using sterile mini‐Gracey curettes (#11–12; Hu‐Friedy, Chicago, IL, USA). Samples were placed in Eppendorf tubes containing 0.15 mL of TE buffer (10 mM Tris‐HCl, 1 mM EDTA, pH 7.6). Subsequently, 100 µL of 0.5 M NaOH was added to each tube, and the samples were stored at −80°C.

Bacterial profiles were analyzed by checkerboard DNA–DNA hybridization for the detection and quantification of 40 bacterial species.^21^, ^22^

### 2.8 Statistical analysis

#### 2.8.1 Clinical data

The normality of the data distribution was assessed using the Shapiro–Wilk test. Clinical and microbiological parameters were summarized as mean ± standard deviation (SD) or proportions. For each participant, average PD and CAL values, as well as average percentages of sites with visible plaque, gingival bleeding, BOP, and SUP, as well as the percentages of sites with PD ≥ 5 mm and PD ≥ 6 mm were calculated and used in group‐level analyses.

Intragroup comparisons throughout the study (baseline, 3 months, and 1 year) were performed using Friedman and Dunn's multiple comparison tests. Intergroup comparisons at each time point were evaluated using the Mann–Whitney *U* test. Between‐group comparisons of categorical variables, such as individuals achieving or not the endpoint for treatment, sex, and self‐reported adverse events, were performed using the Chi‐square test. A significance level of *p *< 0.05 was considered statistically significant.

#### 2.8.2 Microbiological analysis

##### 2.8.2.1 Checkerboard DNA–DNA hybridization

The subgingival microbiota was evaluated for 40 bacterial species using checkerboard DNA–DNA hybridization. The results were expressed both as mean bacterial counts (×10⁵ per site) and relative proportions of the microbial complexes proposed by Socransky et al^21^ (% of total DNA). For each species, mean values were calculated within participants and then averaged across groups.

Between‐group comparisons of bacterial levels and proportions were performed using the Mann–Whitney *U* test. Within‐group comparisons (baseline vs. 3 months and 1 year) were assessed using the Wilcoxon test. Overall statistical significance was maintained at *p* < 0.05.

##### 2.8.2.2 Microbial network analysis

Co‐occurrence coefficients were calculated based on the bacterial quantified profile using the SparCC program.^23^ Bacterium pairs with SparCC values ≥ 0.3 and ≤ −0.3 with the statistically significant threshold were considered to have a co‐occurrence relationship with a positive and negative correlation, respectively. The thresholds for statistical significance were set at *p *< 0.05 and *q *> 0.1 using PseudoPvals in SparCC and Benjamini–Hochberg's procedure.^24^, ^25^ The median correlation of each pairwise comparison and each correlation were estimated by 10 and 500 iterations.^26^ The networks were visualized using version 2.8 of the Cytoscape software.^27^ The bacterial abundance and the strength of their correlations were depicted by the size of the nodes and the thickness of the edges, respectively, with gray and red edges indicating positive and negative correlations, respectively.

## 3 RESULTS

**FIGURE 1:**
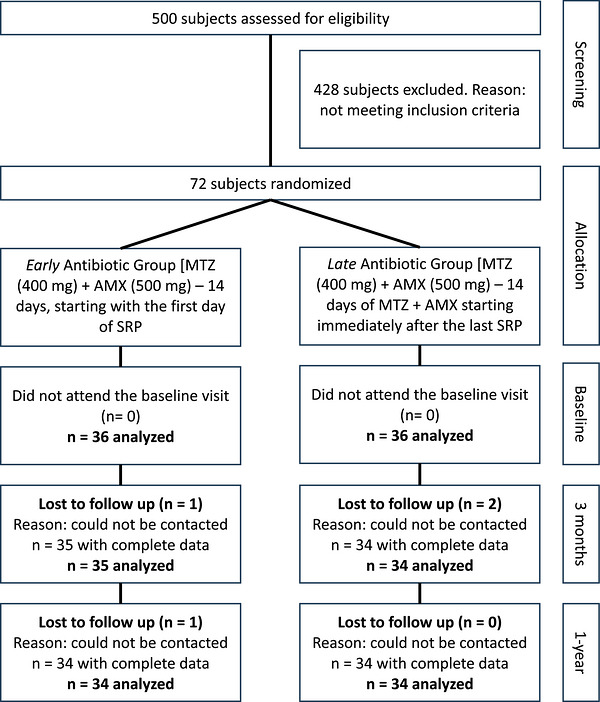
Study flow diagram. AMX, amoxicillin; MTZ, metronidazole; SRP, scaling and root planing.

Recruitment of the participants took place between July, 2012 and October, 2013. Figure 1 presents the study flow chart. In total of 72 participants were enrolled, with 36 assigned to the *early* antibiotic group and 36 to the *late* antibiotic group. Four individuals were lost to follow‐up, and 68 participants were included in the final statistical analysis.

At baseline, the only statistically significant difference between groups was a higher percentage of sites with visible plaque in the *late* group compared with the *early* group. All clinical parameters significantly improved from baseline to 1 year (*p* < 0.05) (Table 1). The only statistically significant difference between groups post‐treatment was a higher percentage of sites with gingival bleeding in the *late* antibiotic group at 3 months. Three patients in the *early* group and two in the *late* group had more than one pill (two or three) remaining in one of the antibiotic/placebo bottles at 14 days. This was considered to have a minor impact, and adherence to the antibiotic protocol was therefore deemed excellent and balanced between groups.

**TABLE 1:** Demographic characteristics and mean (± SD) full‐mouth clinical parameters at baseline and at follow‐up visits.

		Antibiotic moment		
Variable	Time point	Early antibiotic *n* = 34	Late antibiotic *n* = 34	*p*‐value
Sex (male/female)	Baseline	13/21	13/21	1.000
Age (years)	Baseline	42.5 ± 18.6	46.4 ± 17.1	0.357
% sites with				
PD < 4 mm	Baseline	50.1 ± 11.8	47.2 ± 11	0.466
PD 4‐6 mm	Baseline	36.8 ± 7.8	40.4 ± 8.5	0.792
PD ≥ 7 mm	Baseline	13.1 ± 10	12.4 ± 9.1	0.109
PD (mm)	Baseline	4 ± 0.7 a	4 ± 0.5 a	0.514
	3 months	2.6 ± 0.2 b	2.7 ± 0.3 b	0.192
	1 year	2.5 ± 0.3 b	2.6 ± 0.2 b	0.340
	∆ 0–3 months	1.5 ± 0.6	1.4 ± 0.5	0.729
	∆ 0–1 year	1.5 ± 0.7	1.5 ± 0.5	0.878
CAL (mm)	Baseline	4.3 ± 0.8 a	4.4 ± 0.8 a	0.350
	3 months	3 ± 0.6 b	3.3 ± 0.8 b	0.243
	1 year	3 ± 0.6 b	3.3 ± 0.9 b	0.560
	∆ 0–3 months	1.2 ± 0.5	1.1 ± 0.4	0.330
	∆ 0–1 year	1.2 ± 0.5	1.1 ± 0.5	0.573
Visible plaque (% sites)	Baseline	59.4 ± 15.2 a	68.9 ± 15.5 a	**0.008**
	3 months	30.7 ± 16.9 b	36.4 ± 13.9 b	0.151
	1 year	24.7 ± 14.3 b	30.4 ± 17.2 b	0.110
	∆ 0–3 months	28.7 ± 15.9	32.6 ± 14.2	0.175
	∆ 0–1 year	34.7 ± 16	32.5 ± 15.4	0.580
Gingival bleeding (% sites)	Baseline	20.4 ± 16.1 a	27.3 ± 18.1 a	0.053
	3 months	4.4 ± 5.1 b	8.2 ± 7.2 b	**0.006**
	1 year	5.2 ± 4.5 b	6.8 ± 8 b	0.640
	∆ 0–3 months	16 ± 13.8	19.2 ± 16.7	0.277
	∆ 0–1 year	15.2 ± 14.8	20.5 ± 15.9	0.060
BOP (% sites)	Baseline	47 ± 12.8 a	53.3 ± 16.6 a	0.075
	3 months	5.2 ± 6.7 b	7.1 ± 8.6 b	0.061
	1 year	4.5 ± 4.8 b	5.5 ± 5.3 b	0.209
	∆ 0–3 months	41.8 ± 13	46.2 ± 15.7	0.233
	∆ 0–1year	42.5 ± 14	47.9 ± 14.4	0.133
SUP	Baseline	0.4 ± 0.8	0.3 ± 0.8	0.490
	3 months	0 ± 0	0 ± 0	N/A
	1 year	0 ± 0.1	0 ± 0	0.334
	∆ 0–3 months	0.4 ± 0.8	0.3 ± 0.8	0.490
	∆ 0–1 year	0.4 ± 0.8	0.3 ± 0.8	0.802

*Notes*: The significance of differences between baseline and the follow‐up visits was assessed using Friedman and Dunn's multiple comparison tests (different letters indicate significant differences between time points). The significance of differences among groups at each time point was assessed using the Mann–Whitney *U* test (*p*‐value).

Abbreviations: BOP, bleeding on probing; CAL, clinical attachment level; PD, probing depth; SD, standard deviation; SUP, suppuration.

Table 2 presents the number and percentage of participants who achieved the clinical endpoint for treatment (defined as ≤ 4 sites with PD ≥ 5 mm at 1 year), which was the primary outcome variable. No statistically significant differences were observed between the *early* antibiotic and *late* antibiotic groups.

**TABLE 2:** Number and percentage of participants achieving the clinical endpoint^18^ at 3 months and 1 year.

	Treatment group		
Time point	*Early* antibiotic	*Late* antibiotic	*p*‐value
3 months	16 (47.1%)	16 (47.1%)	1
1 year	20 (58.8%)	19 (55.9%)	0.81

*Note*: Chi‐square test (*p*‐value).

The mean number and percentage of sites with a PD ≥ 5 mm, ≥ 6 mm, and ≥ 7 mm at baseline and during follow‐up visits are presented in Table S1 in the online *Journal of Periodontology*. Both groups showed statistically significant reductions in residual sites after treatment, with no significant differences observed between groups at either 3 months or 1 year. No serious adverse events were reported in either group. Likewise, the number and proportion of patients who did not achieve the clinical endpoint^18^ at follow‐up up did not differ between treatments (Table S2 in the online *Journal of Periodontology*).

### 3.1 Microbiological outcomes

**FIGURE 2:**
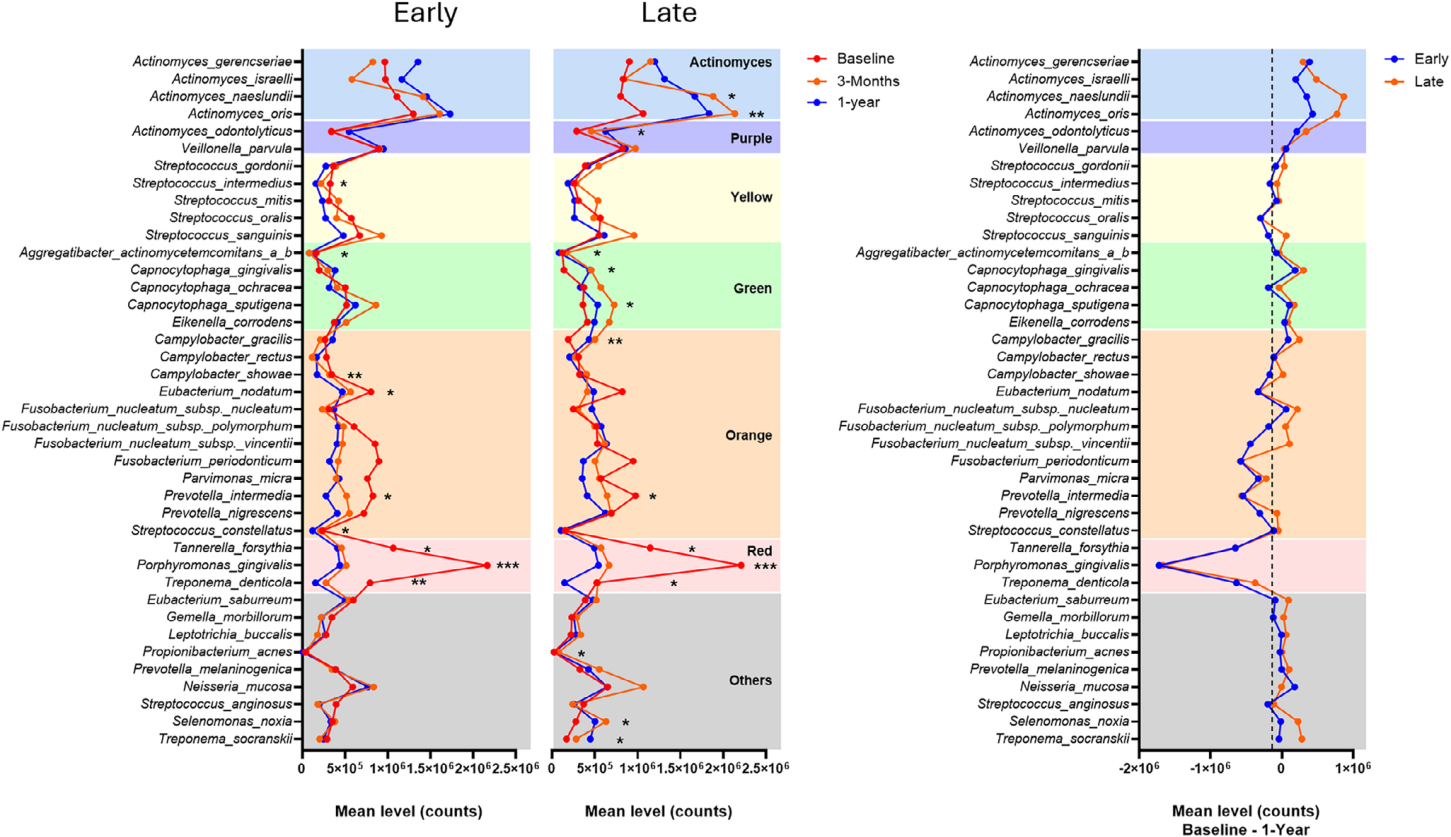
Mean counts of the 40 evaluated bacterial species at baseline, 3 months, and 1 year post‐therapy and mean changes in bacterial levels from baseline to 1 year for each group. Species are ordered according to the microbial complexes proposed by Socransky et al^21^ (1998). Intragroup changes from baseline to 1 year were analyzed using the Wilcoxon signed‐rank test, with significance adjusted for 40 multiple comparisons (*p < 0.05, **p < 0.01, ***p < 0.001). Intergroup differences in mean count changes from baseline to 1 year were assessed using the Mann–Whitney *U* test, with adjustment for baseline values. No statistically significant differences were observed between treatment groups.

Figure 2 presents the mean changes in individual bacterial counts over the course of the study, as well as mean changes in bacterial levels from baseline to 1 year within each treatment group. The three pathogens from the red complex (*Tannerella forsythia*, *Porphyromonas gingivalis*, and *Treponema denticola*), along with *Aggregatibacter actinomycetemcomitans* serotypes *a* and *b*, were significantly reduced at 1 year post‐treatment in both treatment protocols (*p* < 0.05). In addition, patients who received antibiotics earlier showed reductions in four putative pathogens from the orange complex at 1 year compared with baseline (*Campylobacter showae*, *Eubacterium nodatum*, *Prevotella intermedia*, and *Streptococcus constellatus*), whereas in the *late* antibiotic group, only *P. intermedia* was reduced, while *Campylobacter gracilis* increased (*p* < 0.05). Additionally, five species from health‐associated complexes increased at 1 year in the *late* group (*Actinomyces naeslundii*, *Actinomyces oris*, *Capnocytophaga gingivalis*, *Capnocytophaga sputigena*, and *Schaalia odontolytica*—formerly *Actinomyces odontolyticus*), as did *Streptococcus intermedius* in the *early* group (*p* < 0.05). The comparative analysis of changes on individual species counts from baseline to 1 year revealed no statistically significant differences between the *early* and *late* antibiotic groups.

Among participants who failed to reach the clinical endpoint for treatment (Figure S2), both groups showed similar reductions in *P. gingivalis* levels at 1 year. However, only the *early* group exhibited additional decreases in species such as *E. corrodens* and *C. gracilis*.

**FIGURE 3:**
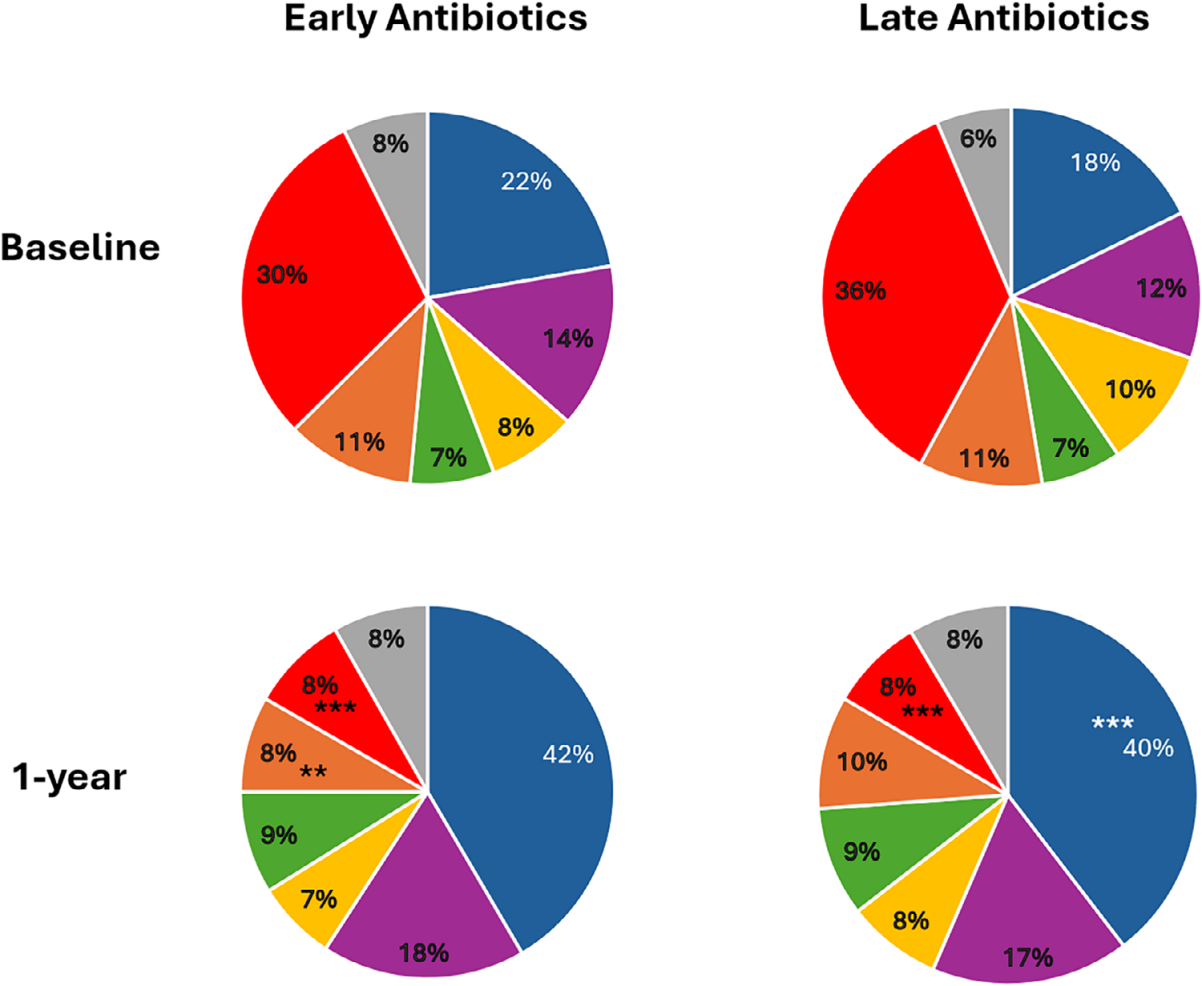
Pie charts presenting the mean proportion of each microbial complex at baseline and 1 year post‐therapy for beginning and ending groups. Colors represent the microbial complexes defined by Socransky et al. (1998).^21^ Within‐group differences between baseline and 1 year post‐therapy were assessed using the Wilcoxon test (***p < 0.001, **p < 0.01*)*. Between‐group differences at each time point were evaluated using the Mann–Whitney *U* test.

Figure 3 presents the mean proportions of microbial complexes at baseline and 1 year post‐therapy for each treatment group. Both groups showed a significant reduction in the proportion of red complex species at 1 year (*p* < 0.001). Additionally, the *early* antibiotic group demonstrated a significant decrease in orange complex species (*p* < 0.01), while the *late* group exhibited a significant increase in *Actinomyces* species (*p* < 0.001).

### 3.2 Network findings

**FIGURE 4:**
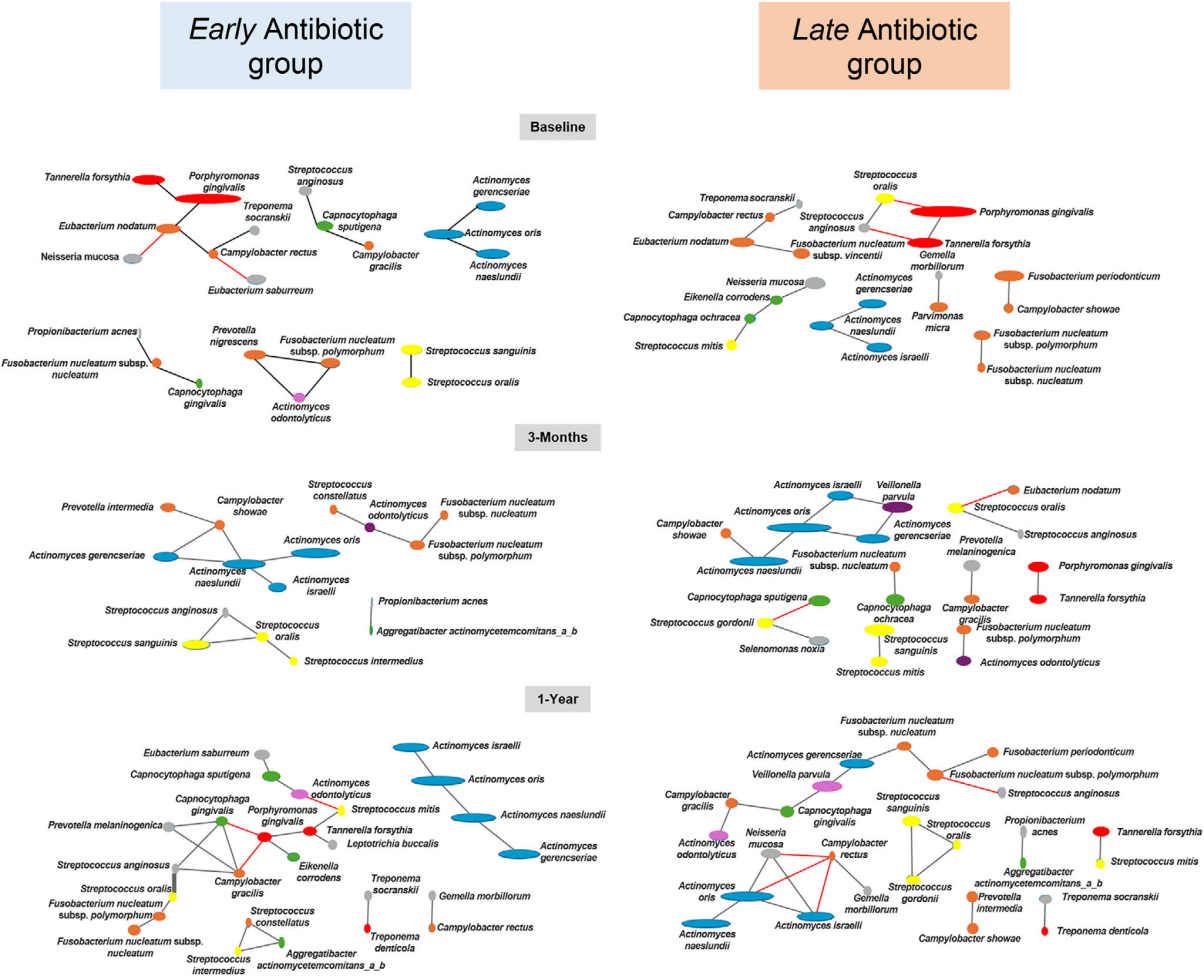
Microbial co‐occurrence network analysis comparing the *early* and *late* antibiotic groups (*n* = 34 per group) at baseline, 3 months, and 1 year post‐treatment. Nodes represent bacterial species, with size proportional to relative abundance and color corresponding to Socransky's microbial complexes.^21^ Edges indicate significant correlations (|r| > 0.3; *p* < 0.05, *q* < 0.1), with gray and red edges denoting positive and negative associations, respectively. Networks were generated using SparCC and visualized in Cytoscape.

The co‐occurrence network analysis showed that both groups exhibited highly connected microbial networks at baseline, primarily dominated by red and orange complex species (Figure 4). By 3 months, both groups showed a reduction in network density and pathogenic clustering. However, red complex species persisted in the *late* antibiotic group, whereas the *early* antibiotic group was characterized by their absence and by the emergence of health‐associated taxa such as *A. naeslundii*, *A. oris*, *A. gerencseriae*, and *S. sanguinis*. These beneficial species occupied integrated positions in the network, reflecting a healthier and more stable ecological shift.

At the 1 year follow‐up, even though red–orange complex organisms are observed in both groups, the *early* group harbors a cluster of four beneficial *Actinomyces* species showing independency and stability. Their lateral and strongly connected position reflects that these *Actinomyces* form a self‐sustaining, protective ecological block that does not rely on pathogenic interactions—a good sign of ecological resilience. In contrast, in the *late* group, *Actinomyces* species remain intricately connected with orange‐complex pathogens such as *Fusobacterium nucleatum* and *Campylobacter rectus*, suggesting a persistent entanglement within a dysbiotic network rather than the establishment of an independent, health‐associated module.

## 4 DISCUSSION

This randomized, double‐blind, placebo‐controlled clinical trial evaluated whether the timing of systemic antibiotic administration relative to SRP influences clinical and microbiological outcomes in patients with stage III/IV periodontitis. The primary outcome, proportion of participants achieving ≤ 4 sites with PD ≥ 5 mm at 1 year,^18^ did not differ significantly between groups. Over half of the participants in both the *early* and *late* antibiotic groups reached this endpoint, suggesting comparable clinical efficacy regardless of the timing of antibiotic administration. These findings are consistent with previous trials demonstrating that adjunctive 500 mg AMX/400 mg MTZ enhances the clinical and microbiological outcomes of mechanical periodontal therapy.^4^, ^17^, ^22^, ^28^, ^29^ It is also important to note that in patients with severe periodontitis treated without antibiotics, the proportion achieving the clinical endpoint typically ranges between 10%–25%.^18^, ^19^, ^28^, ^30^, ^31^ This underscores that both antibiotic protocols tested in this study provided meaningful clinical benefits beyond those expected with SRP alone, with 58.8% and 55.9% of patients in the *early* and *late* antibiotic groups, respectively, achieving the endpoint at 1 year post‐treatment. Both groups showed statistically significant reductions in PD, CAL, BOP, gingival bleeding, and visible plaque from baseline to 1 year. These results support the therapeutic value of adjunctive systemic antibiotics in treating advanced periodontitis. Notably, the only statistically significant difference between groups post‐treatment was a lower percentage of sites with gingival bleeding at 3 months in the *early* antibiotic group, but this difference was no longer present at 1 year. This may suggest an earlier resolution of inflammation in these patients, which could have influenced the microbiological benefits of this treatment. However, given the limited interpretability of gingival bleeding as an outcome measure and the absence of corroborating differences in other clinical parameters, this finding should be interpreted with caution and requires confirmation in future studies.

Despite the lack of statistically significant differences between groups for the clinical parameters, some microbiological distinctions emerged. Participants in the *early* group demonstrated significant reductions in a broader range of periodontal pathogens, including four species of the orange complex at 1 year (C. showae, E. nodatum, S. constellatus, and P. intermedia), whereas only one species of this complex reduced in the *late* group (*P. intermedia*), while another increased (*C. gracilis*). Although these compositional changes suggest delayed microbial remodeling in the *late* group, between‐group comparisons of the overall magnitude of microbial shifts from baseline to 1 year were not statistically significant.

Microbial network analyses highlighted deeper ecological differences between groups. While both began with highly connected dysbiotic networks dominated by red and orange complex species, the *early* antibiotic group showed a faster trajectory of rebiosis, with red complex taxa absent and beneficial species integrated into the network as early as 3 months. By 1 year, some red and orange complex organisms re‐emerged in both groups, which is expected given that patients who did not reach the clinical endpoint are included in the analysis. Nevertheless, the *early* antibiotic group retained a distinct advantage, with its network reorganized around a self‐sustaining cluster of *Actinomyces* spp. and other health‐associated taxa, reflecting resilience and independence from pathogenic hubs. In contrast, the *late* group maintained intricate connections between *Actinomyces* and orange‐complex species such as *F. nucleatum* and *Campylobacter rectus*, suggesting that ecological stability remained compromised. These findings indicate that the timing of antibiotic administration may influence not only short‐term pathogen suppression but also the pace and completeness of microbial rebiosis, favoring the establishment of protective communities when initiated early.

Among participants who failed to reach the clinical endpoint (Figures S1–S4 in the online *Journal of Periodontology*), both groups showed similar reductions in *P. gingivalis* levels. However, only the *early* antibiotic group exhibited additional decreases in species such as *E. corrodens* and *C. gracilis*, suggesting a slightly broader microbial response. This supports the hypothesis that early exposure to systemic antibiotics may enhance subgingival microbiota resolution, even in more treatment‐resistant cases. Nevertheless, it remains unclear whether these microbial shifts have long‐term clinical relevance or merely reflect transient alterations in community composition.^12^, ^32^

Taken together, these results suggest that while both antibiotic protocols are effective, initiating systemic antibiotics at the onset of SRP may accelerate microbial reorganization and promote a more favorable and stable subgingival environment. The beginning of periodontal debridement may represent a critical therapeutic window for modulating the subgingival microbiota. Although clinical outcomes were comparable at 1 year, the microbiological and ecological data point to subtle advantages of early antibiotic administration, particularly in suppressing pathogenic clusters and enhancing the integration of health‐associated species.

There is wide variability in metronidazole dosage and antibiotic duration across studies.^4^ A few RCTs directly compared different durations, showing similar outcomes between 3 and 7 days.^30^, ^31^ and greater benefits with 7 days compared with 3 days.^33^ However, Borges et al (2017)^17^ demonstrated clear advantages for a 14 day regimen in patients with severe periodontitis. They also found a slight benefit for 400 mg over 250 mg metronidazole, with no increase in adverse events. More recently, a machine learning model confirmed total daily metronidazole dose and treatment duration as key predictors of favorable outcomes at 1 year.^34^ Based on this evidence, we have been adopting 500 mg amoxicillin + 400 mg metronidazole TID for 14 days in our studies.

A key strength of this study is its randomized, double‐blind design, as well as the balanced allocation and retention across treatment groups. Notably, this is the first clinical trial to directly compare two systemic antibiotic protocols commonly used in periodontal practice, isolating the effect of timing while holding dosage, duration, and drug choice constant. The integration of clinical and microbiological data and network‐based analyses provides a comprehensive view of treatment outcomes and supports the study's translational relevance.

One limitation is the lack of deeper functional or mechanistic insight into microbial activity. While checkerboard DNA–DNA hybridization allowed reliable species‐level quantification, the absence of transcriptomic, metagenomic, or metabolomic analyses limits interpretation of microbial behavior, host interaction, and resilience. Future studies should incorporate such approaches to better understand the biological relevance of ecological shifts observed with different antibiotic timing strategies. Additionally, radiographs were not available during the study period, preventing radiographic assessment and, therefore, formal disease grading.

In conclusion, the findings of the present study suggest that the timing of antibiotic administration relative to SRP did not influence overall clinical efficacy. Both *early* and *late* antibiotic protocols provided clinical benefits beyond those normally achieved with SRP alone. Notably, initiating antibiotic therapy early may support a more favorable trajectory of microbial rebiosis, contributing to the establishment of a more stable, health‐associated subgingival community over time. These results may help to guide clinical decision‐making, and future studies should incorporate longitudinal and functional analyses (e.g., metagenomics/meta‐transcriptomics) to elucidate the long‐term impact of antibiotic timing on microbial stability and treatment outcomes.

## AUTHOR CONTRIBUTIONS

**Marcelo de Faveri**: Substantial contributions to conception and design of the study; involved in data collection; involved in data interpretation and drafting the manuscript. **Magda Feres**: Substantial contributions to conception and design of the study; involved in data interpretation and drafting the manuscript. **Daiane Fermiano**: Involved in data collection; involved in data interpretation and drafting the manuscript. **Maria Josefa Mestnik**: Involved in data collection. **Luciene C. Figueiredo**: Involved in data collection. **Rafael Oliveira Dias**: Involved in data collection. **Eduardo de Souza Oliveira**: Involved in data analysis; involved in data interpretation and drafting the manuscript. **Takahiko Shiba**: Involved in data analysis. **Shunsuke Matsumura**: Involved in data analysis. **Helio Doyle**: Involved in data analysis. **Edson de Sousa**: Involved in data interpretation and drafting the manuscript. All authors have revised the manuscript critically and have given final approval of the version to be published.

## CONFLICT OF INTEREST STATEMENT

The authors declare no potential conflicts of interest with respect to the authorship and/or publication of this article.

## Supporting information



Supporting Information

Supporting Information

Supporting Information

Supporting Information

Supporting Information

Supporting Information

## References

[1] Mombelli A , Schmid B , Rutar A , Lang NP . Persistence patterns of *Porphyromonas gingivalis*, *Prevotella intermedia/nigrescens*, and *Actinobacillus actinomyetemcomitans* after mechanical therapy of periodontal disease. J Periodontol. 2000;71:14‐21. doi:10.1902/jop.2000.71.1.14 10695934 10.1902/jop.2000.71.1.14

[2] Duarte PM , Feres M , Yassine LLS , et al. Clinical and microbiological effects of scaling and root planing, metronidazole and amoxicillin in the treatment of diabetic and non‐diabetic subjects with periodontitis: a cohort study. J Clin Periodontol. 2018;45:1326‐1335. doi:10.1111/jcpe.12994 30076615 10.1111/jcpe.12994

[3] Sampaio E , Rocha M , Figueiredo LC , et al. Clinical and microbiological effects of azithromycin in the treatment of generalized chronic periodontitis: a randomized placebo‐controlled clinical trial. J Clin Periodontol. 2011;38:838‐846. doi:10.1111/j.1600‐051X.2011.01766.x 21770996 10.1111/j.1600-051X.2011.01766.x

[4] Teughels W , Feres M , Oud V , Martín C , Matesanz P , Herrera D . Adjunctive effect of systemic antimicrobials in periodontitis therapy: a systematic review and meta‐analysis. J Clin Periodontol. 2020;47:257‐281. doi:10.1111/jcpe.13264. **Suppl 22**. 10.1111/jcpe.13264 31994207

[5] van Winkelhoff AJ , Tijhof CJ , de Graaff J . Microbiological and clinical results of metronidazole plus amoxicillin therapy in *Actinobacillus actinomycetemcomitans*‐associated periodontitis. J Periodontol. 1992;63:52‐57. doi:10.1902/jop.1992.63.1.52 1313103 10.1902/jop.1992.63.1.52

[6] Guerrero A , Griffiths GS , Nibali L , et al. Adjunctive benefits of systemic amoxicillin and metronidazole in non‐surgical treatment of generalized aggressive periodontitis: a randomized placebo‐controlled clinical trial. J Clin Periodontol. 2005;32:1096‐1107. doi:10.1111/j.1600‐051X.2005.00814.x 16174275 10.1111/j.1600-051X.2005.00814.x

[7] Herrera D , Alonso B , Leon R , Roldan S , Sanz M . Antimicrobial therapy in periodontitis: the use of systemic antimicrobials against the subgingival biofilm. J Clin Periodontol. 2008;35:45‐66. doi:10.1111/j.1600‐051X.2008.01260.x 18724841 10.1111/j.1600-051X.2008.01260.x

[8] Feres M , Retamal‐Valdes B , Fermiano D , et al. Microbiome changes in young periodontitis patients treated with adjunctive metronidazole and amoxicillin. J Periodontol. 2021;92:467‐478. doi:10.1002/JPER.20‐0128 32844406 10.1002/JPER.20-0128

[9] Feres M , Figueiredo LC , Soares GM , Faveri M . Systemic antibiotics in the treatment of periodontitis. Periodontology 2000. 2015;67:131‐186. doi:10.1111/prd.12075 25494600 10.1111/prd.12075

[10] Mombelli A . Microbial colonization of the periodontal pocket and its significance for periodontal therapy. Periodontology 2000. 2018;76:85‐96. doi:10.1111/prd.12147 29193304 10.1111/prd.12147

[11] Eickholz P , Koch R , Göde M , et al. Clinical benefits of systemic amoxicillin/metronidazole may depend on periodontitis stage and grade: an exploratory sub‐analysis of the ABPARO trial. J Clin Periodontol. 2023;50:1239‐1252. doi:10.1111/jcpe.13838 37293896 10.1111/jcpe.13838

[12] Hagenfeld D , Kleine Bardenhorst S , Matern J , et al. Long‐term changes in the subgingival microbiota in patients with stage III‐IV periodontitis treated by mechanical therapy and adjunctive systemic antibiotics: a secondary analysis of a randomized controlled trial. J Clin Periodontol. 2023;50:1101‐1112. doi:10.1111/jcpe.13824 37160709 10.1111/jcpe.13824

[13] Haffajee AD , Teles RP , Socransky SS . The effect of periodontal therapy on the composition of the subgingival microbiota. Periodontology 2000. 2006;42:219‐258. doi:10.1111/j.1600‐0757.2006.00191.x 16930312 10.1111/j.1600-0757.2006.00191.x

[14] Teles RP , Haffajee AD , Socransky SS . Microbiological goals of periodontal therapy. Periodontology 2000. 2006;42:180‐218. doi:10.1111/j.1600‐0757.2006.00192.x 16930311 10.1111/j.1600-0757.2006.00192.x

[15] Socransky SS , Haffajee AD . Dental biofilms: difficult therapeutic targets. Periodontology 2000. 2002;28:12‐55. doi:10.1034/j.1600‐0757.2002.280102.x 12013340 10.1034/j.1600-0757.2002.280102.x

[16] Papapanou PN , Sanz M , Buduneli N , et al. Periodontitis: consensus report of workgroup 2 of the 2017 World Workshop on the classification of periodontal and peri‐implant diseases and conditions. J Clin Periodontol. 2018;45:S162‐S170. doi:10.1111/jcpe.12946. **Suppl 20**. 29926490 10.1111/jcpe.12946

[17] Borges I , Faveri M , Figueiredo LC , et al. Different antibiotic protocols in the treatment of severe chronic periodontitis: a 1‐year randomized trial. J Clin Periodontol. 2017;44:822‐832. doi:10.1111/jcpe.12721 28303587 10.1111/jcpe.12721

[18] Feres M , Retamal‐Valdes B , Faveri M , et al. Proposal of a clinical endpoint for periodontal trials: the treat‐to‐target approach. J Int Acad Periodontol. 2020;22:41‐53. 32224549

[19] Feres M , Soares GMS , Mendes JAV , et al. Metronidazole alone or with amoxicillin as adjuncts to non‐surgical treatment of chronic periodontitis: a 1‐year double‐blinded, placebo‐controlled, randomized clinical trial. J Clin Periodontol. 2012;39:1149‐1158. doi:10.1111/jcpe.12004 23016867 10.1111/jcpe.12004

[20] Mestnik MJ , Feres M , Figueiredo LC , et al. The effects of adjunctive metronidazole plus amoxicillin in the treatment of generalized aggressive periodontitis: a 1‐year double‐blinded, placebo‐controlled, randomized clinical trial. J Clin Periodontol. 2012;39:955‐961. doi:10.1111/j.1600‐051X.2012.01932.x 22882646 10.1111/j.1600-051X.2012.01932.x

[21] Socransky SS , Haffajee AD , Cugini MA , Smith C , Kent RLJr . Microbial complexes in subgingival plaque. J Clin Periodontol. 1998;25:134‐144. doi:10.1111/j.1600‐051x.1998.tb02419.x 9495612 10.1111/j.1600-051x.1998.tb02419.x

[22] Mestnik MJ , Feres M , Figueiredo LC , Duarte PM , Lira EAG , Faveri M . Short‐term benefits of the adjunctive use of metronidazole plus amoxicillin in the microbial profile and in the clinical parameters of subjects with generalized aggressive periodontitis. J Clin Periodontol. 2010;37:353‐365. doi:10.1111/j.1600‐051X.2010.01538.x 20447259 10.1111/j.1600-051X.2010.01538.x

[23] Friedman J , Alm EJ . Inferring correlation networks from genomic survey data. PLoS Comput Biol. 2012;8:e1002687. doi:10.1371/journal.pcbi.1002687 23028285 10.1371/journal.pcbi.1002687 PMC3447976

[24] Shiba T , Watanabe T , Kachi H , et al. Distinct interacting core taxa in co‐occurrence networks enable discrimination of polymicrobial oral diseases with similar symptoms. Sci Rep. 2016;6:30997. doi:10.1038/srep30997 27499042 10.1038/srep30997 PMC4976368

[25] Komatsu K , Shiba T , Takeuchi Y , et al. Discriminating microbial community structure between peri‐implantitis and periodontitis with integrated metagenomic, metatranscriptomic, and network analysis. Front Cell Infect Microbiol. 2020;10:596490. doi:10.3389/fcimb.2020.596490 33425781 10.3389/fcimb.2020.596490 PMC7793907

[26] Katagiri S , Ohsugi Y , Shiba T , et al. Homemade blenderized tube feeding improves gut microbiome communities in children with enteral nutrition. Front Microbiol. 2023;14:1215236. doi:10.3389/fmicb.2023.1215236 37680532 10.3389/fmicb.2023.1215236 PMC10482415

[27] Kohl M , Wiese S , Warscheid B . Cytoscape: software for visualization and analysis of biological networks. Methods Mol Biol. 2011;696:291‐303. doi:10.1007/978‐1‐60761‐987‐1_18 21063955 10.1007/978-1-60761-987-1_18

[28] Soares GM , Mendes JA , Silva MP , et al. Metronidazole alone or with amoxicillin as adjuncts to non‐surgical treatment of chronic periodontitis: a secondary analysis of microbiological results from a randomized clinical trial. J Clin Periodontol. 2014;41:366‐376. doi:10.1111/jcpe.12217 24834504 10.1111/jcpe.12217

[29] Cosgarea R , Jepsen S , Heumann C , et al. Clinical, microbiological, and immunological effects of 3‐ or 7‐day systemic antibiotics adjunctive to subgingival instrumentation in patients with aggressive (Stage III/IV Grade C) periodontitis: a randomized placebo‐controlled clinical trial. J Clin Periodontol. 2022;49:1106‐1120. doi:10.1111/jcpe.13676 35781888 10.1111/jcpe.13676

[30] Cosgarea R , Juncar R , Heumann C , et al. Non‐surgical periodontal treatment in conjunction with 3 or 7 days systemic administration of amoxicillin and metronidazole in severe chronic periodontitis patients. A placebo‐controlled randomized clinical study. J Clin Periodontol. 2016;43:767‐777. doi:10.1111/jcpe.12559 27027501 10.1111/jcpe.12559

[31] Cosgarea R , Heumann C , Juncar R , et al. One year results of a randomized controlled clinical study evaluating the effects of non‐surgical periodontal therapy of chronic periodontitis in conjunction with three or seven days systemic administration of amoxicillin/metronidazole. PLoS One. 2017;12:e0179592. doi:10.1371/journal.pone.0179592 28662049 10.1371/journal.pone.0179592 PMC5491014

[32] Bizzarro S , Laine ML , Buijs MJ , et al. Microbial profiles at baseline and not the use of antibiotics determine the clinical outcome of the treatment of chronic periodontitis. Sci Rep. 2016;6:20205. doi:10.1038/srep20205 26830979 10.1038/srep20205 PMC4735321

[33] Boia S , Boariu M , Baderca F , et al. Clinical, microbiological and oxidative stress evaluation of periodontitis patients treated with two regimens of systemic antibiotics, adjunctive to non‐surgical therapy: a placebo‐controlled randomized clinical trial. Exp Ther Med. 2019;18:5001‐5015. doi:10.3892/etm.2019.7856 31819766 10.3892/etm.2019.7856 PMC6895779

[34] Feher B , de Souza Oliveira EH , Mendes Duarte P , Werdich AA , Giannobile WV , Feres M . Machine learning‐assisted prediction of clinical responses to periodontal treatment. J Periodontol. 2025. doi:10.1002/JPER.24‐0737 10.1002/JPER.24-0737 PMC12671691 40254962

