# Supplementary figures and images for: Does timing of systemic antibiotics influence periodontal treatment outcomes? A randomized clinical trial
Source: J Periodontol. 2026 Feb 7;97(7):1395–406. doi: 10.1002/jper.70057 (PMC13380390; doi:10.1002/jper.70057)

**SUPPLEMENTARY MATERIAL**

**
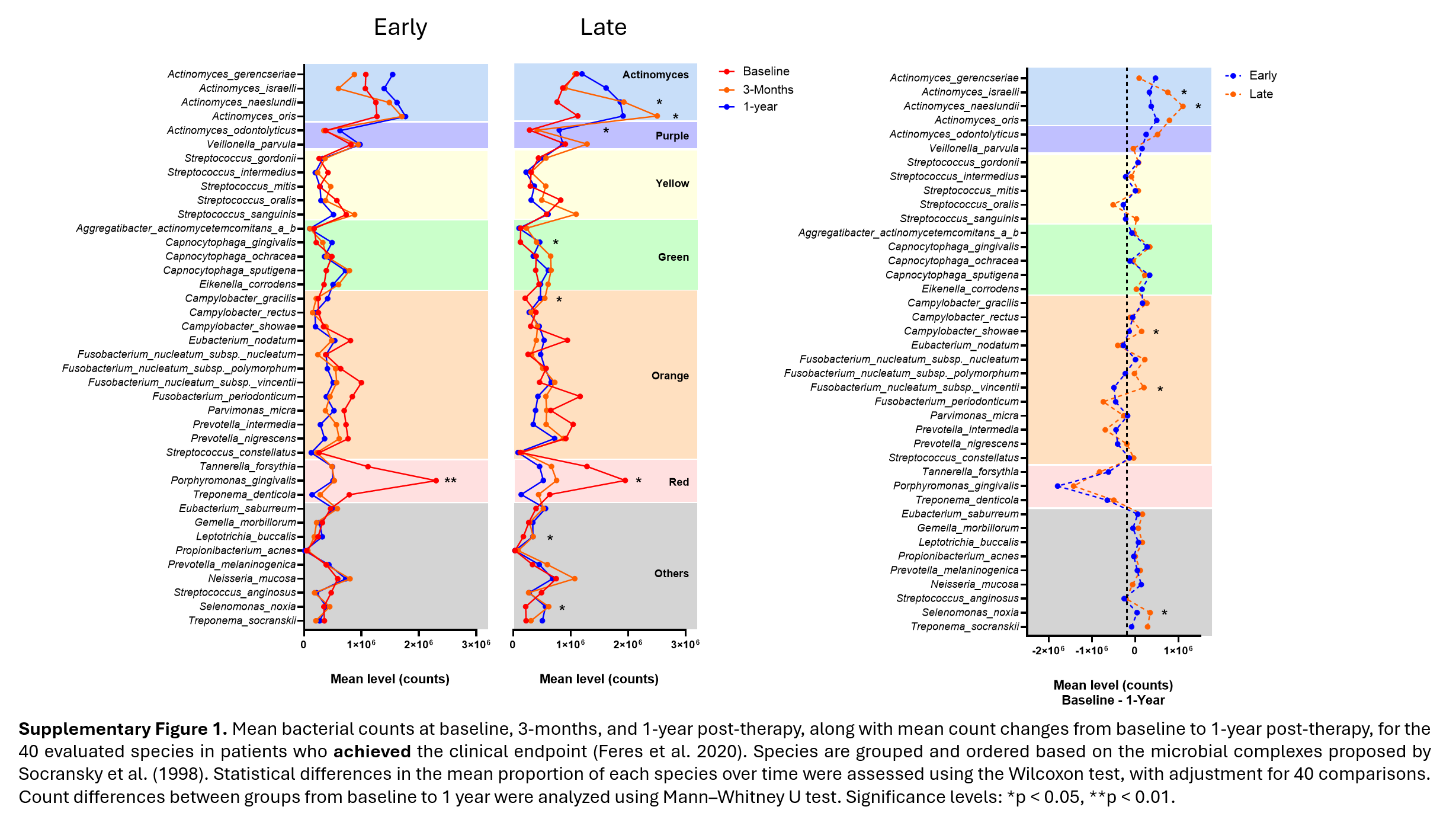
**

Supplement: Supplementary file 1 — Supporting Information [file JPER-97-1395-s005.docx]

**SUPPLEMENTARY MATERIAL**

**
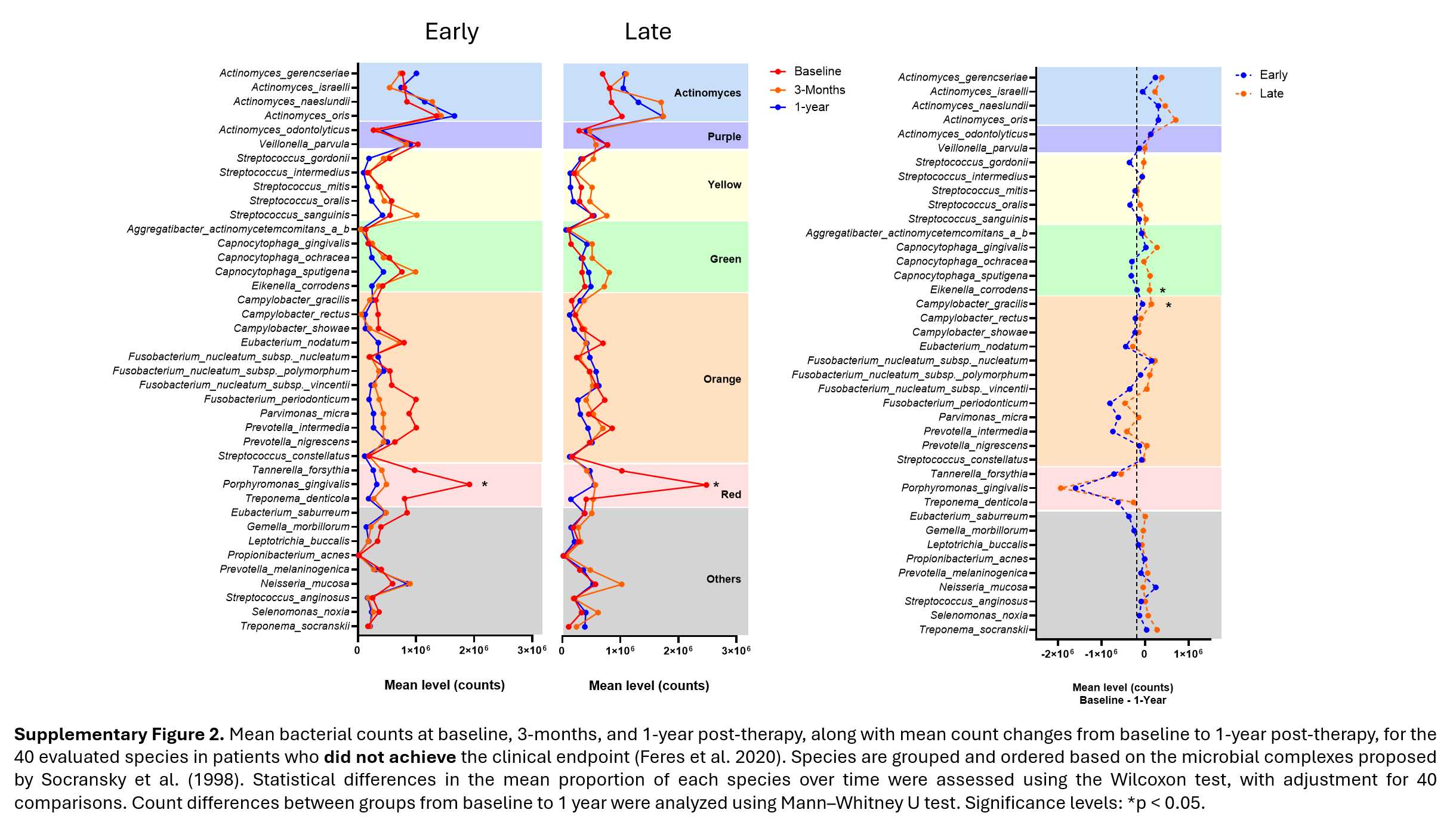
**

Supplement: Supplementary file 2 — Supporting Information [file JPER-97-1395-s001.docx]

**SUPPLEMENTARY MATERIAL**


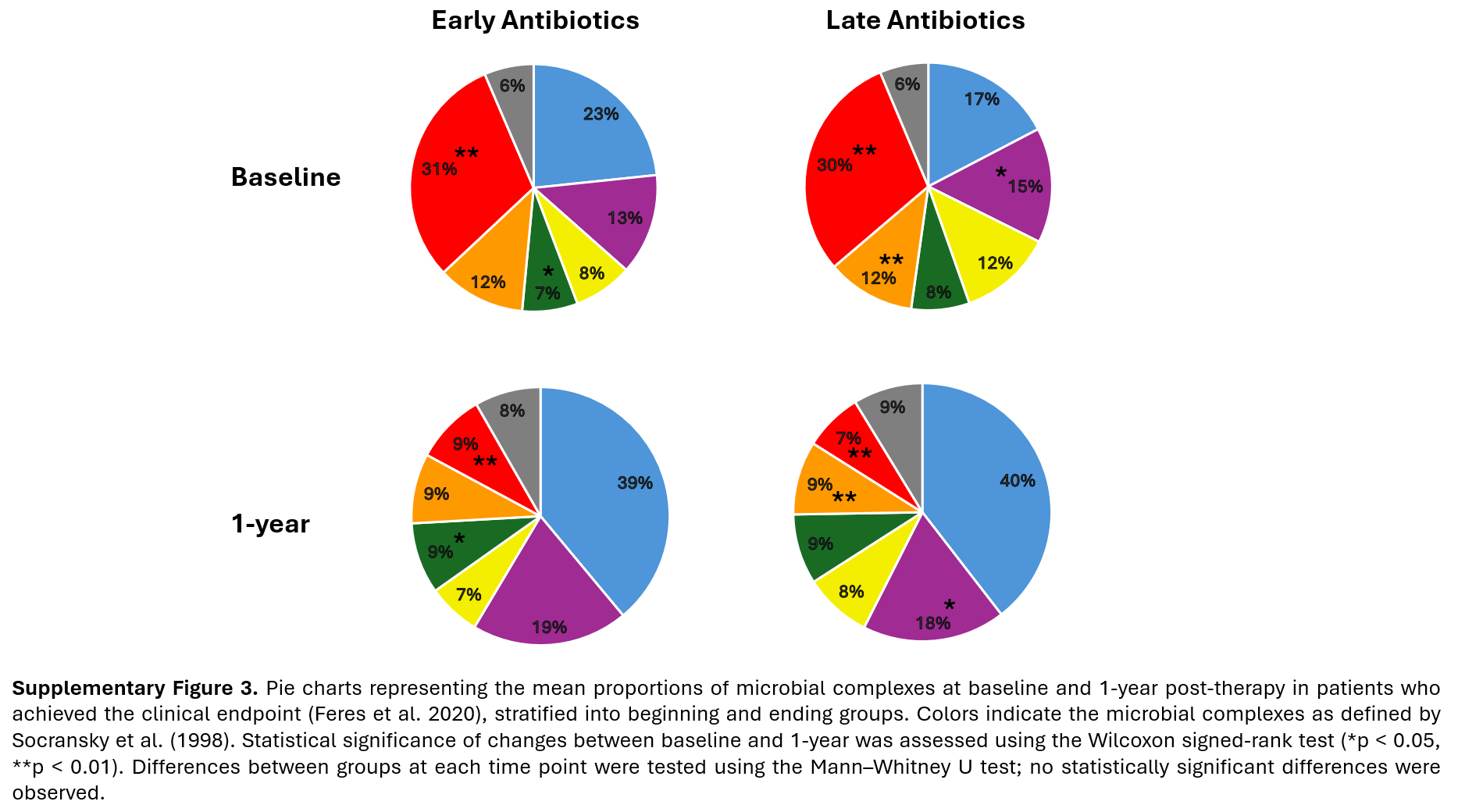

Supplement: Supplementary file 3 — Supporting Information [file JPER-97-1395-s003.docx]

**SUPPLEMENTARY MATERIAL**


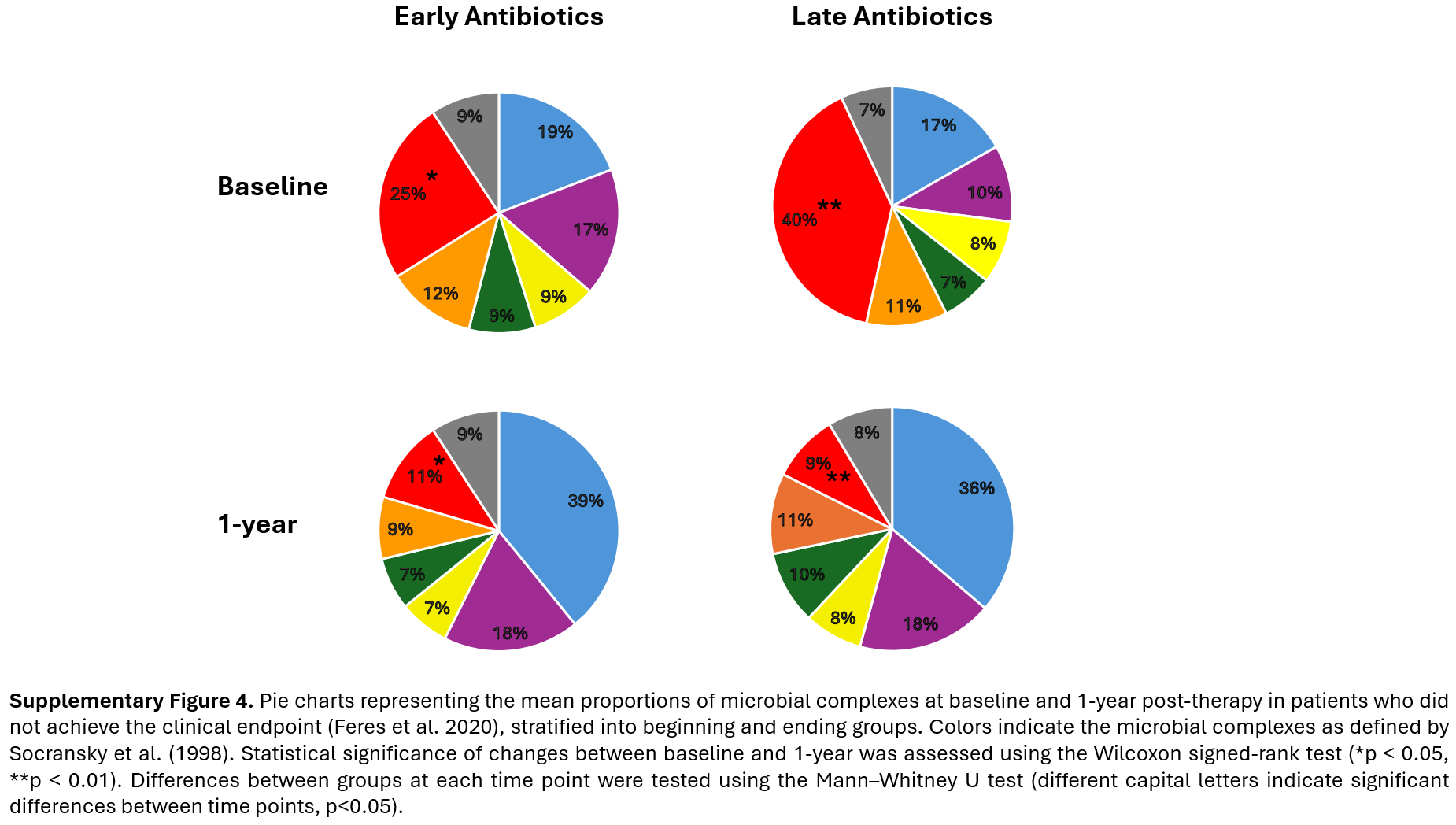

Supplement: Supplementary file 4 — Supporting Information [file JPER-97-1395-s004.docx]
