# Supplementary material for: Does timing of systemic antibiotics influence periodontal treatment outcomes? A randomized clinical trial
Source: J Periodontol. 2026 Feb 7;97(7):1395–406. doi: 10.1002/jper.70057 (PMC13380390; doi:10.1002/jper.70057)
Supplement: Supplementary file 5 — Supporting Information [file JPER-97-1395-s006.docx]

**SUPPLEMENTARY MATERIAL**

| **Supplementary Table 1**. Mean number and percentage (± SD) of sites with PD≥5, ≥6 and ≥7mm at baseline and at follow-up visits post-treatment. | | | | |
| --- | --- | --- | --- | --- |
|  |  | **Treatment groups** | |  |
| **PD category** | **Time point** | ***Early* Antibiotic** | ***Late* Antibiotic** | **^†^p-value** |
|  |  | **Mean # ± SD (mean %)** | **Mean # ± SD (mean %)** |  |
| PD≥5mm | Baseline | 51.5 ± 17.6 (34.7 ± 12.8) a | 54.3 ± 19.3 (37.3 ± 11.8) a | 0.655 |
|  | 3-months | 5.4 ± 4.4 (3.6 ± 3) b | 5.9 ± 5.3 (4.1 ± 3.7) b | 0.847 |
|  | 1-year | 4.9 ± 6.5 (3.3 ± 4.2) b | 5.2 ± 4.6 (3.7 ± 3.2) b | 0.841 |
|  | ∆ 0-3-months | 46.1 ± 18.1 (31.1 ± 13) | 48.4 ± 18.1 (33.2 ± 11.3) | 0.912 |
|  | ∆ 0-1-year | 46.6 ± 19 (31.5 ± 13.6) | 49.1 ± 19.9 (33.6 ± 12.3) | 0.719 |
|  |  |  |  |  |
| PD≥6mm | Baseline | 31.9 ± 17.3 (21.6 ± 11.9) a | 30.7 ± 17.6 (21.3 ± 11.6) a | 0.716 |
|  | 3-months | 2 ± 2.5 (1.3 ± 1.7) b | 1.7 ± 2.8 (1.2 ± 2.2) b | 0.814 |
|  | 1-year | 1.1 ± 2 (0.8 ± 1.4) b | 1.2 ± 1.7 (0.9 ± 1.2) b | 0.602 |
|  | ∆ 0-3-months | 29.9 ± 17.7 (20.3 ± 12) | 29.1 ± 17.3 (20.1 ± 11.4) | 0.927 |
|  | ∆ 0-1-year | 30.7 ± 17.8 (20.8 ± 12.2) | 29.5 ± 17.3 (20.4 ± 11.4) | 0.942 |
|  |  |  |  |  |
| PD≥7mm | Baseline | 19.4 ± 15.4 (13.1 ± 10) a | 17.8 ± 13.7 (12.4 ± 9.1) a | 0.778 |
|  | 3-months | 0.5 ± 1.4 (0.3 ± 0.9) b | 0.6 ± 1.6 (0.5 ± 1.3) b | 0.797 |
|  | 1-year | 0.4 ± 1 (0.3 ± 0.7) b | 0.4 ± 0.9 (0.3 ± 0.6) b | 0.890 |
|  | ∆ 0-3-months | 18.9 ± 15.5 (12.8 ± 10.1) | 17.2 ± 13.7 (11.9 ± 9.1) | 0.846 |
|  | ∆ 0-1-year | 19 ± 15.4 (12.9 ± 10.1) | 17.4 ± 13.8 (12.1 ± 9.2) | 0.535 |
|  |  |  |  |  |
| The significance of differences between baseline and the follow-up visits was assessed using Friedman and Dunn’s multiple comparison tests (different lowercase letters indicate significant differences between time points). The significance of differences among groups at each time point was assessed using the Mann–Whitney U test(†p-value) (different small letters indicate significant differences between pairs of groups). *Abbreviations: PD: probing depth; SD: standard deviation.* | | | | |
