# Supplementary material for: Does timing of systemic antibiotics influence periodontal treatment outcomes? A randomized clinical trial
Source: J Periodontol. 2026 Feb 7;97(7):1395–406. doi: 10.1002/jper.70057 (PMC13380390; doi:10.1002/jper.70057)
Supplement: Supplementary file 6 — Supporting Information [file JPER-97-1395-s002.docx]

**SUPPLEMENTARY MATERIAL**

| **Supplementary Table 2.** Number and percentage of patients who did not achieve the clinical endpoint (Feres et al. 2020) at follow-up visits post-treatment. | | | | |
| --- | --- | --- | --- | --- |
| **Periodontitis Diagnosis** | **Time point** | **Treatment groups** | | **p-value*** |
|  |  | ***Early* antibiotic n=26** | ***Late* antibiotic n=21** |  |
| **Stage III** | 3M | 15 (57.7%) | 9 (42.9%) | 0.311775 |
|  | 1Y | 10 (38.5%) | 9 (42.9%) | 0.735785 |
|  |  | ***Early* antibiotic n=8** | ***Late* antibiotic n=13** |  |
| **Stage IV** | 3M | 3 (37.5%) | 9 (69.2%) | 0.153607 |
|  | 1Y | 3 (37.5%) | 6 (46.2%) | 0.581989 |
| *Chi-square test. |  |  |  |  |
